# Heterologous expression of *Brucella abortus *GroEL heat-shock protein in *Lactococcus lactis*

**DOI:** 10.1186/1475-2859-5-14

**Published:** 2006-03-23

**Authors:** Anderson Miyoshi, Luis G Bermúdez-Humarán, Luciana A Ribeiro, Yves Le Loir, Sérgio C Oliveira, Philippe Langella, Vasco Azevedo

**Affiliations:** 1 Instituto de Ciências Biológicas, Universidade Federal de Minas Gerais, Belo Horizonte – MG, Brasil; 2 Unité d'Ecologie et Physiologie du Sistème Digestif, Institut National de la Recherche Agronomique, Jouy en Josas Cedex, France; 3 Laboratoire de Microbiologie, Institut National de la Recherche Agronomique, Rennes Cedex, France

## Abstract

**Background:**

*Brucella abortus *is a facultative intracellular pathogen that mainly infects cattle and humans. Current vaccines rely on live attenuated strains of *B. abortus*, which can revert to their pathogenic status and thus are not totally safe for use in humans. Therefore, the development of mucosal live vaccines using the food-grade lactic acid bacterium, *Lactococcus lactis*, as an antigen delivery vector, is an attractive alternative and a safer vaccination strategy against *B. abortus*. Here, we report the construction of *L. lactis *strains genetically modified to produce *B. abortus *GroEL heat-shock protein, a candidate antigen, in two cellular locations, intracellular or secreted.

**Results:**

Only the secreted form of GroEL was stably produced in *L. lactis*, suggesting a detrimental effect of GroEL protein when intracellularly produced in this bacterium. Only trace amounts of mature GroEL were detected in the supernatant fraction of induced lactococcal cultures, and the GroEL precursor remained stacked in the cell fraction. Attempts to raise the secretion yields were made, but even when GroEL was fused to a synthetic propeptide, secretion of this antigen was not improved.

**Conclusion:**

We found that *L. lactis *is able to produce, and to secrete, a stable form of GroEL into the extracellular medium. Despite the low secretion efficiency of GroEL, which suggest that this antigen interacts with the cell envelope of *L. lactis*, secretion seems to be the best way to achieve both production and protein yields, regardless of cellular location. The *L. lactis *strain secreting GroEL has potential for *in vivo *immunization.

## Background

*Brucella abortus*, a facultative intracellular Gram-negative bacterium, is the causative agent of brucellosis: a worldwide zoonosis that causes abortion and infertility in cattle, as well as undulant fever, arthritis, endocarditis, meningitis and osteomyelitis in humans [1]; therefore it constitutes both an economic and a public health problem [2]. Current brucellosis vaccines are composed of live attenuated *B. abortus *strains (e.g. S19, RB51). However, most of them present major drawbacks that limit the control of brucellosis: (i) they are pathogenic for humans; (ii) they cause abortion when administered to pregnant females; and (iii) they interfere with the diagnosis of infected animals [2-5]. There is thus a need for the development of more effective and safer vaccines to better control brucellosis.

Current strategies for the development of new vaccines against *B. abortus *have been based on the identification of immunodominant antigens able to elicit a cellular immune response [6-9], which is required to resist the intracellular location of this pathogen [10-12]. GroEL, a well-known heat-shock protein present in various pathogens, can elicit humoral and cellular immune responses in different host models [13-15]. Moreover, cattle and mice infected with *B. abortus *exhibit an immune response to GroEL [8,16,17]. Taken together, these observations suggest the potential of GroEL as a candidate antigen for the development of a brucellosis vaccine.

Recently, new strategies to develop vaccines against infectious diseases have been reported [18-20]. As most pathogenic microorganisms initiate infection through mucosal surfaces, recent approaches have focused on mucosal immunization [21]. A variety of live, attenuated-bacterial strains have been used as carriers to deliver foreign antigens to mammalian hosts [22-25]; however, they still retain invasiveness and virulence properties that limit their use in humans. Lactic acid bacteria (LAB) are promising candidates for the development of new, safe, mucosal vaccines, and different from attenuated pathogenic bacteria, they are food-grade, non-invasive and non-pathogenic organisms [26,27]. Mucosal immunization with genetically modified LAB to produce bacterial and viral antigens has been shown to elicit an immune response [28-32]. We have been particularly interested in the development of a new mucosal vaccine against brucellosis using *Lactococcus lactis*, the model LAB, as a delivery vector. Moreover, as *L. lactis *is a non-commensal and transient bacterium in the digestive tract, induction of immunotolerance to an *L. lactis-*associated antigen is diminished.

We previously reported targeted production (i.e. in the cytoplasm, in the cell wall or into the extracellular medium) of the immunogenic *B. abortus *ribosomal protein L7/L12 in *L. lactis *[33]. Oral administration of a *L. lactis *strain producing a cytoplasmic form of L7/L12 induced partial protection against this pathogen in mice [29]. We have now cloned the *B. abortus groEL *into three different nisin-inducible expression vectors [33,34] for production of GroEL in two cellular locations, intracellular and secreted. Only the secreted form of GroEL was stably produced in *L. lactis*. This new strain has potential for use in vaccination programs to prevent brucellosis.

## Results and discussion

### Construction of recombinant L. lactis strains to produce either cytoplasmic or secreted forms of GroEL

As the protective response depends on the antigen, the delivery system and the location of the antigen [25,35,36], we evaluated the impact of *B. abortus *GroEL production by *L. lactis *in two different cellular locations, intracellular and secreted. Two expression vectors were initially constructed, pCYT:*groEL *and pSEC:*groEL *for cytoplasmic and secreted GroEL production, respectively. These plasmids are derived from two broad-host-range expression vectors, pCYT:Nuc and pSEC:Nuc (Table 1; [34]); pCYT:Nuc harbors a transcriptional fusion between the ribosome-binding site (RBS_*usp*45_) of the *usp45 *gene [37] and the DNA sequence encoding the mature part of the staphylococcal nuclease, NucB [38] (Table 1), and pSEC:Nuc harbors a transcriptional fusion between RBS_*usp*45 _and the DNA sequence encoding the signal peptide (SP_*usp*45_) of Usp45 plus *nucB *(Table 1). In both cases, *nucB *expression is under the control of the nisin-inducible promoter, P_*nisA *_[39].

**Figure 1:**
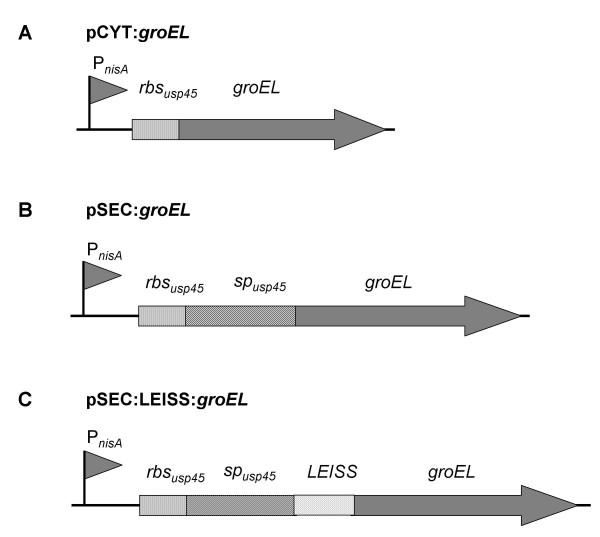
**Schematic representation of expression cassettes for controlled and targeted GroEL production in *L. lactis***. For details of plasmid constructions, see the text and Table 1. P_*nisA*_: nisin-inducible promoter; *rbs*_*Usp*45_: ribosome binding site of Usp45 gene; *sp*_*Usp*45_: DNA sequence encoding the signal peptide of Usp45 gene; *LEISS*: DNA sequence encoding the LEISSTCDA synthetic propeptide; *groEL*: *B. abortus groEL *coding sequence (not to scale).

The vector to target GroEL protein in the cytoplasm of *L. lactis*, pCYT:*groEL*, was obtained as follows: A 1641-bp DNA fragment encoding GroEL was PCR-amplified from the vector pMal-GroEL (Table 1; [17]). Two oligonucleotides, containing two restriction sites, were designed on the basis of the genomic DNA sequence from the *B. abortus groEL *gene (Genbank accession number M82975): i) CTF*groEL *for the coding strand: 5'- GGATGCATGCTGCAAAAGACGTA -3', in which the *Nsi*I site is underlined; and ii) CTR*groEL *for the complementary strand: 5'- CGGAATTCTTAGAAGTCCATGCC -3', in which the *Eco*RI site is underlined. The resulting amplified product was treated with *Nsi*I and *EcoR*I and then cloned into purified backbone isolated from *Nsi*I-*EcoR*I-cut pCYT:Nuc expression vector, replacing the DNA sequence encoding for NucB (Table 1; Figure 1A). To obtain the vector to target GroEL protein to the extracellular medium (i.e. secreted) of *L. lactis*, pSEC:*groEL*, the following procedures were adopted: The *groEL *gene was PCR amplified from pMal-GroEL (Table 1; [17]). The oligonucleotides were: i) SCF*groEL *for the coding strand: 5'- GGATGCAT**CA**GCTGCAAAAGACGTA -3', in which the *Nsi*I site is underlined and CA (in bold) was added to adapt the reading frame of *sp*_*Usp45*_; and ii) SCR*groEL *for the complementary strand: 5'- CGGTTAACTTAGAAGTCCATGCC -3', in which the *Hpa*I site is underlined. The PCR product was then digested by *Nsi*I and *Hpa*I and cloned into purified backbone isolated from *Nsi*I-*Eco*RV-cut pSEC:Nuc expression vector, again replacing the DNA sequence encoding for NucB (Table 1; Figure 1B).

**Table 1:** Bacterial strains and plasmids used in this work

Strain/plasmid	Relevant characteristics	Source
Bacterial strains		
*E. coli *TG1	*supE*, *hsd*, *Δ5*, *thi*, *Δ *(*lac-proAB*), F'(*traD36 proAB-lacZΔM15*)	[50]
*L. lactis *NZ9000	*L. lactis *subsp. *cremoris *(derivative strain of MG1363, carrying *nisRK *genes on the chromosome)	[51]
Plasmids		
pMal-GroEL	pMal expression vector carrying the *B. abortus groEL *gene	[17]
pCYT:Nuc	pWV01/Cm^r^; expression vector containing the fusion *rbs*_*Usp*45_::*nucB*, under the control of P_*nisA*_	[34]
pSEC:Nuc	pWV01/Cm^r^; expression vector containing the fusion *rbs*_*Usp*45_::*sp*_*Usp*45_::*nucB*, under the control of P_*nisA*_	[34]
pSEC:LEISS:Nuc	pWV01/Cm^r^; expression vector containing the fusion *rbs*_*Usp*45_::*sp*_*Usp*45_::*LEISS*::*nucB*, under the control of P_*nisA*_	[33]
pCYT:groEL	pWV01/Cm^r^; plasmid containing the fusion *rbs*_*Usp*45_::*groEL*, under the control of P_*nisA*_	This work
pSEC:groEL	pWV01/Cm^r^; plasmid containing the fusion *rbs*_*Usp*45_::*sp*_*Usp*45_::*groEL*, under the control of P_*nisA*_	This work
pSEC:LEISS:groEL	pWV01/Cm^r^; plasmid containing the fusion *rbs*_*Usp*45_::*sp*_*Usp*45_::*LEISS*::*groEL*, under the control of P_*nisA*_	This work

In both cases, pCYT:*groEL *and pSEC:*groEL *were first obtained in *E. coli *TG1 and then transferred into *L. lactis *NZ9000 [40,41]. All constructions were confirmed by DNA sequencing. Surprisingly, during procedures to recover *L. lactis *NZ9000 colonies harboring the pCYT:*groEL *plasmid, we observed that such colonies did not grow normally, taking around four days to be visible in selective M17-agar plates; while *L. lactis *NZ9000 colonies harboring the pSEC:*groEL *plasmid, hereafter called NZ(pSEC:*groEL*), normally grew in 18–24 hours. Moreover, when some colonies were grown in selective liquid medium, they reached a maximum OD_600nm _of around 0.05, after overnight culture, compared to OD_600nm _of around 1.5 for NZ(pSEC:*groEL*). One hypothesis to explain this phenomenon is that the pCYT:*groEL *plasmid has a basal expression level, and GroEL could therefore interact with lactococcal proteins, generating detrimental disorders in the host cellular metabolism. Prokaryotic chaperones are functionally well conserved and, once cloned in a foreign host, a chaperone may interact with the host proteins [42,43]. We already observed similar phenomena in *L. lactis *with various viral, prokaryote and eukaryote proteins (for a review see [44]); lactococcal strains engineered to produce cytoplasmic protein forms had a reduced growth rate, and consequent absent or low levels of heterologous protein production.

### L. lactis is able to produce a stable secreted form of GroEL

**Figure 2:**
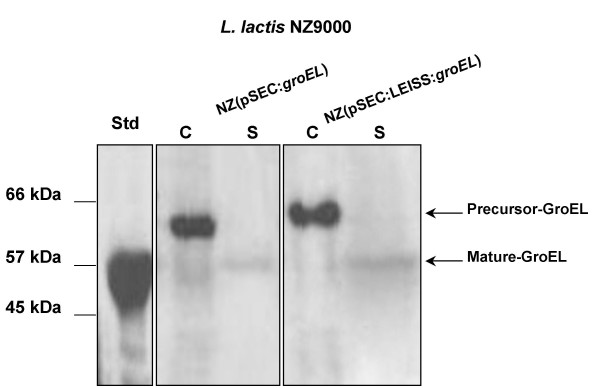
**Western blot analyses of nisin-induced *L. lactis *NZ9000 (pSEC:*groEL*) and (pSEC:LEISS:*groEL*) strains**. Protein extracts of culture samples of NZ(pSEC:*groEL*) and NZ(pSEC:LEISS:*groEL*) strains were prepared from cell (lanes C) and supernatant (lanes S) fractions and were analyzed by Western blotting using anti-GroEL antibodies. The migration positions of precursor- and mature-GroEL forms are indicated by arrows. Purified GroEL (around 12 μg) was used as the standard (lane Std), and molecular masses are indicated on the left.

As we did not succeeded in obtaining an *L. lactis *strain that produced GroEL in the cytoplasm, we continued the analysis of the *L. lactis *strain that produced a secreted form of GroEL. To evaluate whether this recombinant strain is able to produce and export GroEL outside the cell, we performed Western blot analysis of proteins extracted from cell (C) and supernatant (S) fractions of induced and non-induced NZ(pSEC:*groEL*) cultures. Analysis of induced NZ(pSEC:*groEL*) samples revealed only one band in the C fraction, with an expected size of around 60 kDa, which corresponds to the GroEL precursor (SP_*Usp45*_::GroEL) (Figure 2). In the S fraction, we also detected only one band at the expected size for mature GroEL (around 57 kDa) (Figure 2). However, in this latter case, only trace amounts of mature GroEL could be detected in the S fraction; this indicates that the GroEL precursor remained stacked in the C fraction, probably associated with the cell envelope. Thus, secreted form of GroEL seems not to be interfering with the host physiology due to the fact that GroEL is fused to SP_*Usp45 *_which in turn targets the hybrid protein to the extracellular medium, or at least, in this case, to the cell envelope. SP_*Usp45*_::GroEL might undergo rapid folding right after their synthesis, which interferes with (or hampers) the secretion process. Moreover, sometimes, secreted proteins require subsequent folding and maturation steps to acquire their active conformation. The secretion efficiency (SE; the ratio of mature protein secreted in the supernatant as a fraction of intracellular content) was estimated to be ~3–5%. Inefficiency in *B. abortus *GroEL secretion was previously reported by Leclerq et al. (2002; [17]), using the mammalian expression vector pCMV-tPA, containing a signal peptide sequence fused to *groEL*. Very low levels of GroEL were observed both in the C and S fractions from D17 cells (dog osteosarcoma cell line), in spite of the presence of GroEL transcripts. In this case, a possible explanation is that as *B. abortus *GroEL is able to associate with the bacterial surface through the type IV secretion system and to interact directly or indirectly with cellular prion protein (PrP^C^) on host cells [45], these properties may interfere with the secretion process. On the other hand, in the case of *L. lactis*, low GroEL SE may be due to an interaction with the cell envelope, mediated by a secretion system other than the type IV system, which is not present in lactococcal cells [46]. A similar effect was already observed in a *L. lactis *strain designed to produce a secreted form of bovine rotavirus nonstructural protein 4 (NSP4) [47]. No NSP4 was detected in the S fraction, and both precursor and mature protein were only detected in the C fraction. The authors suggest that NSP4 could be associated with the cell envelope, probably due to hydrophobic domains that prevent its release into the medium.

Interestingly, the secreted GroEL seems not to be a target for the unique *L. lactis *housekeeping extracellular protease (HtrA; [48]), since degradation products were not detected by Western blotting (see Methods section) in the S fraction of induced NZ(pSEC:*groEL*) cultures samples (Figure 2). As previously reported [48,49], a number of exported heterologous proteins already produced in wild type *L. lactis *strains are recognized by this protease as foreign, being degraded during translocation steps across the cell envelope. We suppose that this did not occur in our strain because *B. abortus *GroEL seems to be structurally and functionally well-conserved. Comparison analyses of GroEL amino acid sequence from *B. abortus *S19 (accession number: AAA22997) and *L. lactis *IL1403 (accession number: NP266550) showed that these sequences possess around 54% identity, and both sequences harbor a highly-conserved Cpn60 chaperonin motif.

In conclusion, even though low SE was observed, *L. lactis *was able to produce and target *B. abortus *GroEL to the extracellular medium. Moreover, degradation products related to lactococcal HtrA activity were not observed in the S fraction from induced NZ(pSEC:*groEL*) culture samples, and so *L. lactis *seems to be able to produce a stable form of GroEL.

### Synthetic propeptide does not enhance secretion efficiency of GroEL

Previous studies showed that the synthetic propeptide LEISSTCDA (hereafter called LEISS) can enhance SE of heterologous proteins in *L. lactis *(for a review see [44]). We examined whether LEISS could improve SE of GroEL. For this purpose, we used pSEC:LEISS vector, which is a derivative of pSEC:Nuc vector, plus a DNA fragment encoding for LEISS synthetic propeptide fused between SP_*Usp*45 _and *nucB *(Table 1; [33]). The DNA fragment encoding GroEL was cloned into pSEC:LEISS, using the same experimental procedure as that used for cloning the secreted form of GroEL (see above). The resulting plasmid, pSEC:LEISS:*groEL *(Table 1; Figure 1C), was established in *L. lactis *NZ9000 [NZ(pSEC:LEISS:*groEL*)] and GroEL production and secretion was then examined by Western blot analysis. LEISS did not exert any significant influence on SE of GroEL, since comparable amounts of GroEL were present in the S fraction from both NZ(pSEC:*groEL*) and NZ(pSEC:LEISS:*groEL*) (Figure 2). Note that GroEL is the second reported protein in which LEISS has no influence on the SE. The first one was the hybrid protein Nuc-NSP4 [49].

## Conclusion

This work is part of an ongoing project geared to producing and testing new *B. abortus *antigens that could be used as alternative vaccines against brucellosis. Here we have described the construction of lactococcal strains that produce *B. abortus *GroEL heat-shock protein, a well-known immunodominant target for both humoral and cellular immune responses [8,16]. *L. lactis *was able to produce, and to secrete, a stable form of GroEL into the extracellular medium. Despite concerns about the low SE of GroEL, which suggest that this antigen interacts with the cell envelope of *L. lactis*, secretion seems to be the best way to achieve both production and protein yields, regardless of cellular location. Therefore, this new *L. lactis *strain has potential for oral immunization trials. Immunization assays using this strain are now in progress and will allow definition of the immune response and the level of protection that GroEL confers against challenge with *B. abortus*.

## Methods

### Bacterial strains, growth conditions and plasmids

*Escherichia coli *TG1 (Table 1; [50]) was aerobically grown in Luria-Bertani medium at 37°C. *L. lactis *NZ9000 (Table 1; [51]) was grown in M17 medium supplemented with 0.5% glucose (GM17) at 30°C. When required, antibiotics were added as follows: ampicillin (100 μg/ml) and chloramphenicol (10 μg/ml) for *E. coli*, and chloramphenicol (10 μg/ml) for *L. lactis*.

Lactococcal plasmids pCYT:Nuc, pSEC:Nuc and pSEC:LEISS:Nuc (Table 1; [33,34]) were used in order to: i) control the expression of the *B. abortus groEL*, through a nisin-inducible promoter, P_*nisA *_[39]; and ii) target the *B. abortus *GroEL either to the cytoplasm or to the extracellular medium. For further details about plasmid constructions, see the results and discussion section.

### DNA manipulations

General DNA manipulation techniques were carried out according to standard procedures [40]. Unless otherwise indicated, DNA restriction and modification enzymes were used as recommended by the suppliers. DNA fragments were isolated from agarose gels with the Concert™ Rapid Gel Extraction System (Gibco BRL). PCR amplifications were made using Taq DNA polymerase (Invitrogen™) in a DNA thermocycler (MJ Research, Inc.). Plasmid DNA from *E. coli *and *L. lactis *was isolated, as previously described [40,41]. DNA sequencing was carried out on double-stranded plasmid DNA by the dideoxy chain termination method [52] with MegaBACE Sequencing Systems (Amersham Biosciences). Routine amino acid homology searches were performed by the "Basic Local Alignment Search Tool" (BLAST; [53]), service of the National Center for Biotechnology Information (NCBI). Amino acid sequence similarity searches were performed by the ClustalW [54], service of the European Bioinformatics Institute. Further analyses for the identification of protein-conserved motifs were performed with the "Protein Families Database" (Pfam; [55]), service of the Wellcome Trust Sanger Institute.

### Conditions of nisin induction

For induction of the nisin promoter, overnight cultures of recombinant *L. lactis *strains harboring pCYT:*groEL*, pSEC:*groEL *or pSEC:LEISS:*groEL *(Table 1) were used to inoculate fresh medium at a dilution of 1/100. At an optical density at 600 nm (OD_600_) of ~0.4, 1 ng/ml of nisin (Sigma) was added and cultures were incubated for one hour, before performing cell fractionation and protein extractions.

### Protein extractions and Western blotting

Protein samples were prepared from *L. lactis *cultures, as previously described [56], except for the introduction of protease inhibitors and mild precipitation procedures. Briefly, protein samples were prepared from 2 ml of cultures, and the cell pellet and supernatant were treated separately. To inhibit proteolysis in supernatant samples, 1 mM phenylmethylsulfonyl fluoride (PMSF) and 10 mM dithiothreitol (DTT) were added. Proteins were then precipitated by addition of 100 μl of 100% trichloroacetic acid, incubated for 10 min on ice, and then centrifuged 10 min at 17,500 × g at 4°C. For the cell fraction, TES-Lys buffer (25% sucrose, 1 mM EDTA, 50 mM Tris-HCl [pH 8.0], lysozyme [10 mg/ml]) was complemented with 1 mM PMSF and 10 mM DTT. Twelve percent sodium dodecyl sulfate-polyacrylamide gel electrophoresis (SDS-PAGE) and Western blot, using anti-GroEL antibodies [17], were performed, as described previously [40]. Immunodetections were carried out with protein G horseradish peroxidase conjugate (BioRad) and the ECL Kit (Dupont-NEN), as recommended by the suppliers. Quantification of GroEL was performed by scanning blots after immunodetection, comparing the signals to those of known amounts of purified GroEL [38].

## Authors' contributions

AM, LGBH, LAR, YLL and SCO equally contributed to this work, participating in the plasmid and strain constructions, molecular biology procedures, sequence alignments, scientific discussion, data interpretation, and manuscript draft. PL and VA share credit in this work for senior authorship.

## References

[B1] Nicoletti PL, Young EJ, Corbel MJ (1989). Relationship between animal and human disease. Brucellosis: clinical and laboratory aspects.

[B2] Boschiroli M, Foulongne V, O'Callaghan D (2001). Brucellosis: a worldwide zoonosis. Curr Opin Microbiol.

[B3] Corner LA, Alton GG (1981). Persistence of *Brucella abortus *strain 19 infection in adult cattle vaccinated with reduced doses. Res Vet Sci.

[B4] Schurig GG, Roop RM, Bagchi T, Boyle S, Buhrman D, Sriranganathan N (1991). Biological properties of RB51; a stable rough strain of *Brucella abortus*. Vet Microbiol.

[B5] Cheville NF, Stevens MG, Jensen AE, Tatum FM, Halling SM (1993). Immune responses and protection against infection and abortion in cattle experimentally vaccinated with mutant strains of *Brucella abortus*. Am J Vet Res.

[B6] Oliveira SC, Splitter GA (1994). Subcloning and expression of the *Brucella abortus *L7/L12 ribosomal gene and T-lymphocyte recognition of the recombinant protein. Infect Immun.

[B7] Roop RM, Fletcher TW, Sriranganathan NM, Boyle SM, Schurig GG (1994). Identification of an immunoreactive *Brucella abortus *HtrA stress response protein homolog. Infect Immun.

[B8] Oliveira SC, Harms JS, Banai M, Splitter GA (1996). Recombinant *Brucella abortus *proteins that induce proliferation and gamma-interferon secretion by CD4+ T cells from Brucella-vaccinated mice and delayed-type hypersensitivity in sensitized guinea pigs. Cell Immunol.

[B9] Vemulapalli R, He Y, Boyle SM, Sriranganathan N, Schurig GG (2000). Overexpression of protective antigen as a novel approach to enhance vaccine efficacy of *Brucella abortus *strain RB51. Infect Immun.

[B10] Zhan Y, Yang J, Cheers C (1993). Cytokine response of T-cell subsets from Brucella abortus-infected mice to soluble Brucella proteins. Infect Immun.

[B11] Oliveira SC, Splitter GA (1995). CD8+ type 1 CD44hi CD45 RBlo T lymphocytes control intracellular *Brucella abortus *infection as demonstrated in major histocompatibility complex class I- and class II-deficient mice. Eur J Immunol.

[B12] Oliveira SC, Harms JS, Rech EL, Rodarte RS, Bocca AL, Goes AM, Splitter GA (1998). The role of T cell subsets and cytokines in the regulation of intracellular bacterial infection. Braz J Med Biol Res.

[B13] Lin J, Adams LG, Ficht TA (1992). Characterization of the heat shock response in *Brucella abortus *and isolation of the gene encoding the GroEL heat shock protein. Infect Immun.

[B14] Lemos JA, Castro GM (1998). Expression of heat-shock proteins in *Streptococcus pyogenes *and their immunoreactivity with sera from patients with streptococcal diseases. J Med Microbiol.

[B15] Silva CL (1999). The potential use of the heat shock proteins to vaccinate against mycobacterial infections. Microbes Infect.

[B16] Lin J, Adams LG, Ficht TA (1996). Immunological response to the *Brucella abortus *GroEL homolog. Infect Immun.

[B17] Leclerq S, Harms JS, Rosinha GMS, Azevedo V, Oliveira SC (2002). Induction of a Th1-type of immune response but not protective immunity by intramuscular DNA immunisation with *Brucella abortus *GroEL heat-shock gene. J Med Microbiol.

[B18] Shata MT, Stevceva L, Agwale S, Lewis GK, Hone DM (2000). Recent advances with recombinant bacterial vaccine vectors. Mol Med Today.

[B19] Kurstak E (2001). Recent progress in vaccines development and new trends in immunization. Vaccine.

[B20] Ellis RW (2001). Technologies for the design, discovery, formulation and administration of vaccines. Vaccine.

[B21] Cripps AW, Kyd JM, Foxwell R (2001). Vaccines and mucosal immunisation. Vaccine.

[B22] Thole JER, van Dalen PJ, Havenith CEG, Pouwels PH, Seegers JFML, Tielen FD, van dar Zee M, Zegers ND, Shaw M (2000). Live bacterial delivery systems for development of mucosal vaccines. Curr Opin Mol Ther.

[B23] Medina E, Guzmán CA (2001). Use of live bacterial vaccine vectors for antigen delivery: potential and limitations. Vaccine.

[B24] Gentschev I, Dietrich G, Spreng S, Kolb-Maurer A, Brinkmann V, Grode L, Hess J, Kaufmann SHE, Goebel W (2001). Recombinant attenuated bacteria for the delivery of subunit vaccines. Vaccine.

[B25] Mielcarek N, Alonso S, Locht C (2001). Nasal vaccination using live bacterial vectors. Adv Drug Deliv Rev.

[B26] Wells JM, Robinson K, Chamberlain LM, Schofield KM, Le Page RW (1996). Lactic acid bacteria as vaccine delivery vehicles. Antonie Van Leeuwenhoek.

[B27] Pouwels PH, Leer RJ, Shaw M, den Bak-Glashouwer MJH, Tielen FD, Smit E, Martinez B, Jore J, Conway PL (1998). Lactic acid bacteria as antigen delivery vehicles for oral immunization purposes. Int J Food Microbiol.

[B28] Xin KQ, Hoshino Y, Toda Y, Igimi S, Kojima Y, Jounai N, Ohba K, Kushiro A, Kiwaki M, Hamajima K, Klinman D, Okuda K (2003). Immunogenicity and protective efficacy of orally administered recombinant *Lactococcus lactis *expressing surface-bound HIV Env. Blood.

[B29] Pontes DS, Dorella FA, Ribeiro LA, Miyoshi A, Le Loir Y, Gruss A, Oliveira SC, Langella P, Azevedo V (2003). Induction of partial protection in mice after oral administration of *Lactococcus lactis *producing *Brucella abortus *L7/L12 antigen. J Drug Target.

[B30] Robinson K, Chamberlain LM, Lopez MC, Rush CM, Marcotte H, Le Page RW, Wells JM (2004). Mucosal and cellular immune responses elicited by recombinant *Lactococcus lactis *strains expressing tetanus toxin fragment C. Infect Immun.

[B31] Pei H, Liu J, Cheng Y, Sun C, Wang C, Lu Y, Ding J, Zhou J, Xiang H (2005). Expression of SARS-coronavirus nucleocapsid protein in *Escherichia coli *and *Lactococcus lactis *for serodiagnosis and mucosal vaccination. Appl Microbiol Biotechnol.

[B32] Bermudez-Humaran LG, Cortes-Perez NG, Lefevre F, Guimaraes V, Rabot S, Alcocer-Gonzalez JM, Gratadoux JJ, Rodriguez-Padilla C, Tamez-Guerra RS, Corthier G, Gruss A, Langella P (2005). A novel mucosal vaccine based on live lactococci expressing E7 antigen and IL-12 induces systemic and mucosal immune responses and protects mice against Human Papillomavirus Type 16-induced tumors. J Immunol.

[B33] Ribeiro LA, Azevedo V, Le Loir Y, Oliveira SC, Dieye Y, Piard JC, Gruss A, Langella P (2002). Production and targeting of the *Brucella abortus *antigen L7/L12 in *Lactococcus lactis*: first step towards food-grade live vaccines against brucellosis. Appl Environ Microbiol.

[B34] Bermúdez-Humarán LG, Langella P, Commissaire J, Gilbert S, Le Loir Y, L'Haridon R, Corthier G (2003). Controlled intra- or extracellular production of staphylococcal nuclease and ovine omega interferon in *Lactococcus lactis*. FEMS Microbiol Lett.

[B35] Norton PM, Brown HW, Le Page RWF (1994). The immune response to *Lactococcus lactis *: implications for its use as a vaccine delivery vehicle. FEMS Microbiol Lett.

[B36] Norton PM, Brown HW, Wells JM, Macpherson AM, Wilson PW, Le Page RW (1996). Factors affecting the immunogenicity of tetanus toxin fragment C expressed in Lactococcus lactis. FEMS Immunol Med Microbiol.

[B37] van Asseldonk M, Rutten G, Oteman M, Siezen RJ, de Vos WM, Simons G (1990). Cloning of usp45, a gene encoding a secreted protein from *Lactococcus lactis *subsp. *lactis *MG1363. Gene.

[B38] Le Loir Y, Gruss A, Ehrlich SD, Langella P (1994). Direct screening of recombinants in Gram-positive bacteria using the secreted staphylococcal nuclease as a reporter. J Bacteriol.

[B39] de Ruyter PG, Kuipers OP, de Vos WM (1996). Controlled gene expression systems for *Lactococcus lactis *with the food-grade inducer nisin. Appl Environ Microbiol.

[B40] Sambrook J, Fritsch EF, Maniatis T (1989). Molecular cloning: a laboratory manual.

[B41] Langella P, Le Loir Y, Ehrlich SD, Gruss A (1993). Efficient plasmid mobilization by pIP501 in *Lactococcus lactis *subsp. *lactis*. J Bacteriol.

[B42] Fink AL (1999). Chaperone-mediated protein folding. Physiol Rev.

[B43] Brocchieri L, Karlin S (2000). Conservation among HSP60 sequences in relation to structure, function, and evolution. Protein Sci.

[B44] Le Loir Y, Azevedo V, Oliveira SC, Freitas DA, Miyoshi A, Bermúdez-Humarán LG, Nouaille S, Ribeiro LA, Leclercq S, Gabriel JE, Guimaraes VD, Oliveira MN, Charlier C, Gautier M, Langella P (2005). Protein secretion in *Lactococcus lactis *: an efficient way to increase the overall heterologous protein production. Microb Cell Fact.

[B45] Watarai M, Kim S, Erdenebaatar J, Makino S, Horiuchi M, Shirahata T, Sakaguchi S, Katamine S (2003). Cellular prion protein promotes *Brucella *infection into macrophages. J Exp Med.

[B46] Bolotin A, Wincker P, Mauger S, Jaillon O, Malarme K, Weissenbach J, Ehrlich SD, Sorokin A (2001). The complete genome sequence of the lactic acid bacterium *Lactococcus lactis *ssp. *lactis *IL1403. Genome Res.

[B47] Enouf V, Langella P, Commissaire J, Cohen J, Corthier G (2001). Bovine rotavirus nonstructural protein 4 produced by *Lactococcus lactis *is antigenic and immunogenic. Appl Environ Microbiol.

[B48] Poquet I, Saint V, Seznec E, Simoes N, Bolotin A, Gruss A (2000). HtrA is the unique surface housekeeping protease in *Lactococcus lactis *and is required for natural protein processing. Mol Microbiol.

[B49] Miyoshi A, Poquet I, Azevedo V, Commissaire J, Bermúdez-Humarán L, Domakova E, Le Loir Y, Oliveira SC, Gruss A, Langella P (2002). Controlled production of stable heterologous proteins in *Lactococcus lactis*. Appl Environ Microbiol.

[B50] Gibson TJ (1984). Studies on the Epstein-Bart virus genome. PhD thesis.

[B51] Kuipers OP, de Ruyter PG, Kleerebezem M, de Vos WM (1998). Quorum sensing controlled gene expression in lactic acid bacteria. J Biotechnol.

[B52] Sanger F, Nicklen S, Coulson AR (1977). DNA sequencing with chain-terminating inhibitors. Proc Natl Acad Sci USA.

[B53] Basic Local Alignment Search Tool – BLAST. http://www.ncbi.nlm.nih.gov/blast.

[B54] ClustalW. http://www2.ebi.ac.uk/clustalw/.

[B55] Protein Families Database – Pfam. http://www.sanger.ac.uk/Software/ Pfam/search.shtml.

[B56] Le Loir Y, Gruss A, Ehrlich SD, Langella P (1998). A nine-residue synthetic propeptide enhances secretion efficiency of heterologous proteins in *Lactococcus lactis*. J Bacteriol.

